# Identification of the circRNA-miRNA-mRNA Regulatory Network in Bladder Cancer by Bioinformatics Analysis

**DOI:** 10.1155/2021/9935986

**Published:** 2021-11-16

**Authors:** Jiancheng Lv, Ping-an Chang, Xin Li, Xiao Yang, Jie Han, Hao Yu, Zijian Zhou, Haiwei Yang, Pengchao Li, Jiexiu Zhang, Qiang Lu

**Affiliations:** ^1^Department of Urology, The First Affiliated Hospital of Nanjing Medical University, Nanjing 210029, China; ^2^Department of Urology, Dongtai People Hospital, Yancheng 224000, China; ^3^Department of Urology, Graduate School of Medicine, Kyoto University, Kyoto 606-8501, Japan

## Abstract

In recent years, increasing evidence shows that circular RNA (circRNA) disorder is closely related to tumorigenesis and cancer progression. However, the regulatory functions of most circRNAs in bladder cancer (BCa) remain unclear. This study was aimed at exploring the molecular regulatory mechanism of circRNAs in BCa. We obtained four datasets of circRNA, microRNA (miRNA), and messenger (mRNA) expression profiles from the Gene Expression Omnibus and The Cancer Genome Atlas microarray databases and identified 434, 367, and 4799/4841 differentially expressed circRNAs, miRNAs, and mRNAs, respectively. With these differentially expressed RNAs, we established a circRNA-miRNA-mRNA targeted interaction network. A total of 18, 24, and 51 central circRNAs, miRNAs, and mRNAs were identified, respectively. Among them, the top 10 mRNAs that had high connectivity with other circRNAs and miRNAs were regarded as hub genes. We detected the expression levels of these 10 mRNAs in 16 pairs of BCa tissues and adjacent normal tissues through quantitative real-time polymerase chain reaction. The differentially expressed mRNAs and central mRNAs were enriched in the processes and pathways that are associated with the growth, differentiation, proliferation, and apoptosis of tumor cells. The outstanding genes (CDCA4, GATA6, LATS2, RHOB, ZBTB4, and ZFPM2) also interacted with numerous drugs, indicating their potency as biomarkers and drug targets. The findings of this study provide a deep understanding of the circRNA-related competitive endogenous RNA regulatory mechanism in BCa pathogenesis.

## 1. Introduction

Bladder cancer (BCa) is a tumor affecting the mucosa of the bladder and is one of the top 10 diagnosed tumors worldwide [1, 2]. It is the most common malignant tumor of the urinary system, occupying the first place in the incidence of urogenital tumors in China while second only to prostate cancer in the west [2]. BCa can occur regardless of age [3, 4]. The incidence of BCa increases with age, with the highest rate among people aged 50–70 years [4, 5]. With the progress and development of medical technology in recent years, the treatments for BCa are also undergoing continuous improvement and innovation [6, 7]. However, the clinical treatment of BCa is complicated [6] by variable and complex pathogenic factors, including internal genetic factors and external environmental factors [8]. In addition, RNA research showed that the physiological functions of RNA are diverse and complex, related to all genes, cell regulation, and biological activity [9]. These results have also laid a good clinical foundation for advancing RNA-based targeted therapy, which may expand the scope of drug and pharmacological research by screening “drug” targets [9].

Circular RNA (circRNA) plays an important role in the occurrence and development of diseases and can be used as a potential molecular biological marker [10]. circRNA is another endogenous noncoding RNA discovered after microRNA (miRNA) and long-chain noncoding RNA [11]. circRNA is a type of circular molecule formed by trans-splicing to make the 3′ and 5′ ends covalently bonded. It is not easily degraded by exonuclease and is more stable than linear RNA [11]. circRNA has many biological functions. It can combine with miRNA and exert its “sponge” effect to inhibit miRNA function, thereby releasing miRNA-targeted mRNA transcripts. It can also regulate gene transcription and translation through competition with linear splicing [11]. Given its diverse regulatory functions, circRNA can regulate many cancer driver genes and immunotherapy targets [10, 11]. Wu et al. found that has_circ_0002052 is significantly downregulated in osteosarcoma (OS) tissues and cell lines. In their study, the overexpression of hsa_circ_000205 suppresses the expression of the tumor suppressor miRNA miR-1250 and increases the expression of the miR-1250 target gene APC22, which is a negative regulator of the Wnt/*β*-catenin signaling pathway, causing the OS process to slow down [12]. In lung cancer, hsa_circ_0007059 functions as an inhibitory regulator. It activates the Wnt/*β*-catenin and ERK1/2 pathways through the sponge of miR-378, thereby reducing the proliferation of lung cancer cells and EMT [13].

In the current study, we collected four datasets of circRNA, miRNA, and mRNA expression profiles that are related to BCa from the Gene Expression Omnibus (GEO) and The Cancer Genome Atlas (TCGA) databases. Differentially expressed RNAs can be identified through the R software package, which is an effective and convenient bioinformatics method to identify differential correlation genes as potential biomarkers [14]. After predicting the target genes among the differentially expressed circRNAs, miRNAs, and mRNAs, we constructed a circRNA-miRNA-mRNA targeted interaction network and screened the significant central RNAs. We verified the expression of hub genes in eight pairs of BCa tissues by quantitative real time-PCR (qRT-PCR). To evaluate the main functional pathways of BCa, Gene Ontology (GO) annotations and Kyoto Encyclopedia of Genes and Genomes (KEGG) pathway analysis were performed to evaluate the physiological pathways and functions of differential mRNAs and targeted genes. In addition, combined analysis of the correlation with the pathological stage, overall survival, and drug sensitivity verified that the hub genes influence the RNA mechanism of action of BCa. These findings provide a deep understanding of the circRNA-miRNA-mRNA chains driving the pathological mechanism of BCa and provide potential anticancer biomarkers.

## 2. Methods

### 2.1. Data Collection

Relevant data were downloaded from the GEO (https://www.ncbi.nlm.nih.gov/gds) and TCGA (https://tcga-data.nci.nih.gov/tcga/) databases to uncover genes driving the RNA mechanism of action of BCa. The circRNA and mRNA expression datasets (GSE92675 and GSE133624) were downloaded from the GEO database. The miRNA and mRNA expression profiles of TCGA BLCA were downloaded from TCGA database. The total tissue samples with the clinical symptoms were reserved. The samples that had clinical symptoms and were withdrawn were deleted. The primary screening of genes was performed for follow-up analyses.

### 2.2. Differential Expression Analysis

Four datasets of mRNA, miRNA, and circRNA expression profiles were analyzed in this study. To screen effective DEGs, we used log2FC as the evaluative criteria for measuring the clinical samples, namely, the scale standards of the significant differences between tumor and normal tissue and required FDR ≤ 0.05 and Ilog2FCI ≥ 1. Then, we performed the differential expression analysis with all genes (mRNA, miRNA, and circRNA) expressed in each sample to identify the relative differentially expressed genes (DEGs). To verify the significantly changing genes in each group, we performed ANOVA in R to determine the genetic variance between the tumor and normal sample groups. Statistical significance was considered at *P* < 0.05.

### 2.3. Functional Enrichment Analysis

Functional enrichment analysis was performed using the clusterProfiler software, an ontology-based R package for annotating and visualizing the integrated discovery, to understand the biological functions of the screened DEGs. All DEGs were used for the GO and KEGG pathway enrichment analyses. With the cutoff set to *P* < 0.05, the significant biological processes and pathways could be established.

### 2.4. Hub Gene Identification

The differentially expressed circRNAs were uploaded into the search tool (http://bioinformatics.zju.edu.cn/Circ2Disease/search.html) for retrieving the targeted miRNAs. Then, two R packages of multiMiR and miRNAtap were used to predict the targeted mRNAs of all differential miRNAs. We screened the top 50 differential circRNAs, miRNAs, and mRNAs to find the most significant hub genes in BCa development. Then, we merged them to construct a targeted interaction network. Genes with highly interconnected nodes in the network were considered hub genes.

### 2.5. Clinical Sample Acquisition

Sixteen pairs of BCa tumor tissues and adjacent healthy tissues were obtained from patients with BCa who underwent radical cystectomy at the First Affiliated Hospital of Nanjing Medical University from 2015 to 2018. All patients were diagnosed with BCa. All tissue samples were frozen in liquid nitrogen before RNA extraction. The experiment was granted approval by the ethics board at the hospital. All participants signed informed consent.

### 2.6. RNA Isolation and qRT-PCR

We extracted total RNA from tissues through the TRIzol reagent (Invitrogen, USA). Then, cDNA was compounded by reverse transcription with the HiScript II reagent (Vazyme, China) for qRT-PCR. LightCycler 480 (Roche, USA) was used for qRT-PCR verifying hub genes. *β*-Actin served as the control for relative mRNA identification. Each experiment was repeated three times, and the outcomes were calculated using the 2^−ΔCT^ method. All primers used in the experiments were purchased from Tsingke (Beijing, China) and are listed in Supplemental Table 1.

### 2.7. GEPIA

Gene expression profiles, tumor stage, and survival rate were analyzed to reveal the prognostic value of hub genes on patients with BCa. The expression value and clinical data of BCa were downloaded from the GEO and TCGA databases. The total gene groups in the data were of mRNA, miRNA, and circRNA. The correlation between gene expression and stage was determined using GEPIA (http://gepia.cancer-pku.cn/index.html) [15]. The correlation between gene expression and tissues/tumor stage/overall survival was analyzed using the R package limma software. The survival rate was estimated, the statistical significance was analyzed using the Kaplan–Meier method, and statistical significance was considered at *P* < 0.05.

### 2.8. Genetic Pathway Activity Analysis

We explored the pathway activity that interacted with the 22 hub genes and screened for the remarkable pathways that had higher scores than the others and interacted mostly with hub genes.

### 2.9. Drug Sensitivity of Outstanding Genes

The drug sensitivity of the outstanding hub genes was analyzed using GSCALite to provide support for the drug selection of real hub gene-targeted therapy. The drug response data across cancers were obtained from the GDSC and CTRP datasets. Finally, the remarkable genes that showed sensitivity to numerous drugs and small molecules may serve as potential markers for tumor treatment and clinical drug selection.

### 2.10. The Human Protein Atlas (HPA) Analysis

The protein level of 10 hub genes in BCa tissues or normal urinary bladder tissues was determined by HPA analysis (https://www.proteinatlas.org/). The results were presented in the form of immunohistochemistry.

## 3. Results

### 3.1. Identifications of the Coexpression Network of DEGs in BCa

**Figure 1:**
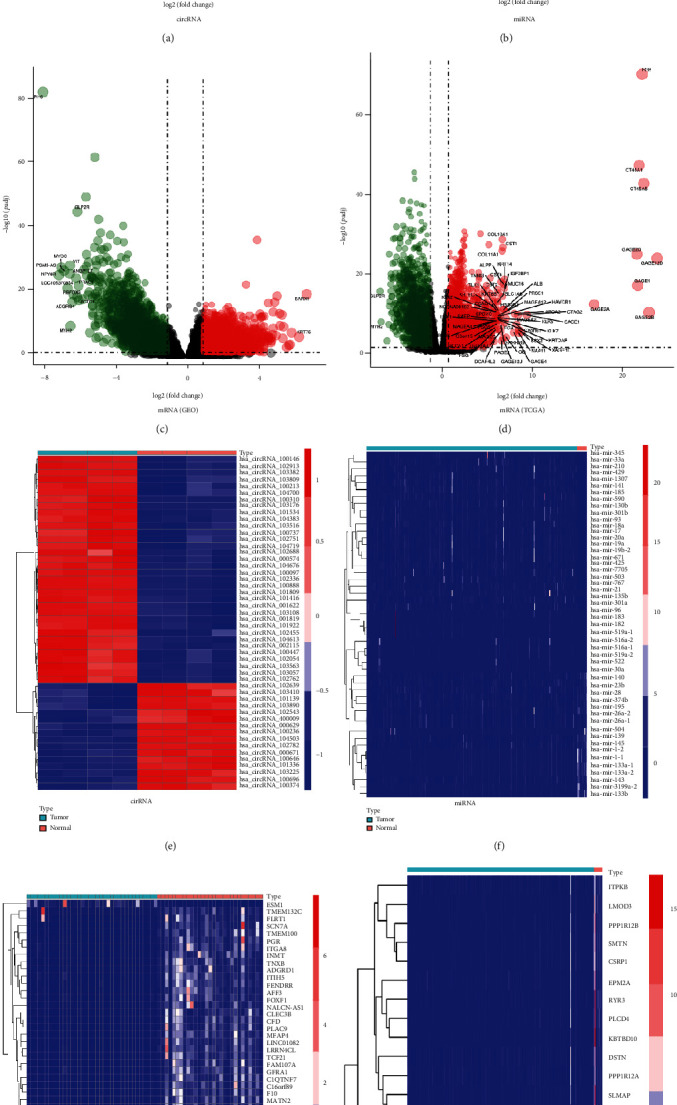
Volcano plots and heat maps of the differentially expressed circRNAs (a, e), miRNAs (b, f), and mRNAs (c, g, d, h) datasets, respectively. The DEGs were identified with the conditions of PFDR < 0.05 and Ilog2FCI ≥ 1. DEGs: differentially expressed genes.

With the conditions of *P*_FDR_ < 0.05 and ∣log_2_FC | ≥1, 434 differentially expressed circRNAs (167 upregulated and 267 downregulated) were identified between the BCa and normal groups. For the miRNA database, 367 differentially expressed miRNAs (269 upregulated and 98 downregulated) were found. For the mRNA dataset from the GEO database, 4799 differentially expressed mRNAs (1903 upregulated DEGs and 2896 downregulated) were detected. In the mRNA dataset from TCGA database, 4841 DEGs (2717 upregulated and 2124 downregulated) were identified (Table 1). We overlapped the mRNAs from TCGA and GSE133624 datasets and identified 2290 overlapped DEGs, including 971 upregulated and 1319 downregulated mRNAs (Table 2). As shown in the corresponding volcano plots and heat maps, the differentially expressed circRNAs, miRNAs, and mRNAs were sufficient to distinguish the BCa group from the normal group (Figures 1(a)–1(h)).

### 3.2. Functional Enrichment Analysis of the DEGs in the Overlapped mRNA Datasets

**Figure 2:**
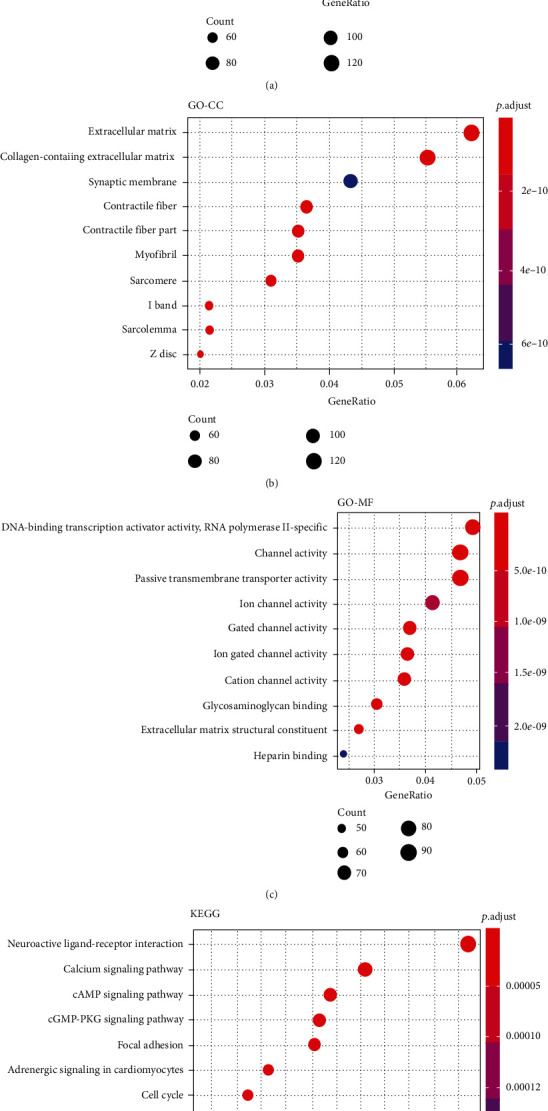
Functional enrichment analysis of the overlapped DGEs by GO and KEGG pathway analysis. (a) Results of the DEGs that were enriched in the biological process by GO analysis. (b) Results of the DEGs that were enriched in the cellular component by GO analysis. (c) Results of the DEGs that were enriched in the molecular function by GO analysis. (d) Results of the DEGs that were enriched in the metabolic pathway by KEGG pathway analysis. GO: Gene Ontology analysis; KEGG: Kyoto Encyclopedia of Genes and Genomes pathways analysis; GO-BP: biological process; GO-CC: cellular component; GO-MF: molecular function.

The biological functions of the differentially expressed mRNAs associated with BCa were explored. The overlapping DEGs were subjected to GO and KEGG pathway analyses to explore the biological functions of the candidate genes. As shown in Figure 2, all of the significant terms in the annotated systems were assigned in different colored dots when compared with other relative significance of the enriched terms. The sizes and depths of the colored dots represented the enrichment gene numbers and differences, respectively. GO enrichment analysis indicated that the DEGs were enriched in the biological processes (GO-BP) with 10 significant terms. The top five enrichment terms were about the muscle system process, skeletal system development, muscle contraction, muscle organ development, and regulation of ion transmembrane transport (Figure 2(a)). In the enrichment of cellular components (GO-CC), 11 significant terms were found. Among them, the extracellular matrix, collagen-containing extracellular matrix, and synaptic membrane were the top three enrichment terms (Figure 2(b)). The DEGs enriched in the molecular functions (GO-MF) included 10 significant terms. The top 5 enrichment terms were about the DNA-binding transcription activator activity, RNA polymerase II-specific channel activity, passive transmembrane transporter activity, and ion and gated channel activities (Figure 2(c)). In addition, KEGG pathway analysis showed that the significant enriched terms were involved in 11 pathways. Among them, the top five enriched terms were about the neuroactive ligand-receptor interaction, calcium signaling pathway, cAMP signaling pathway, cGMP-PKG signaling pathway, and focal adhesion (Figure 2(d)). In a word, the enrichment results of the merging DEGs in BCa indicated the relational physiological function driven by differentially expressed mRNAs. Thus, the effective genes could be further explored and screened as potential biomarkers.

### 3.3. Identification of Universal Hub Genes among the Differentially Expressed RNAs

**Figure 3:**
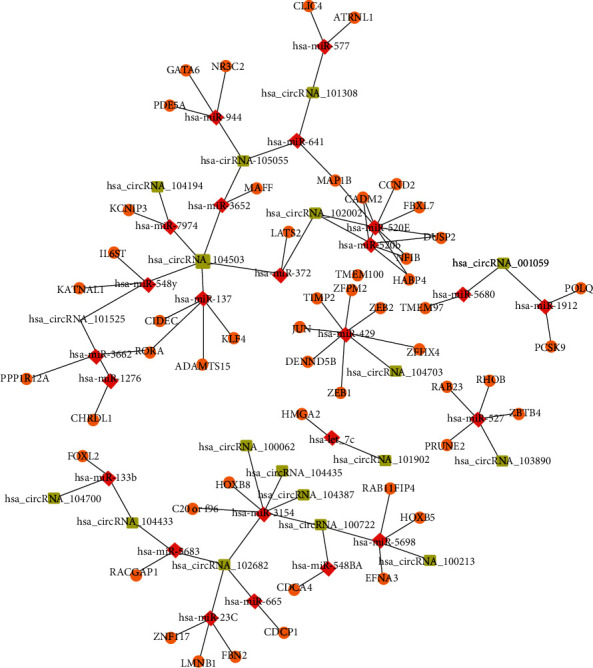
Hub gene identification from the targeted interaction network of the top 50 DEGs (circRNAs, miRNAs, and mRNAs).

**Figure 4:**
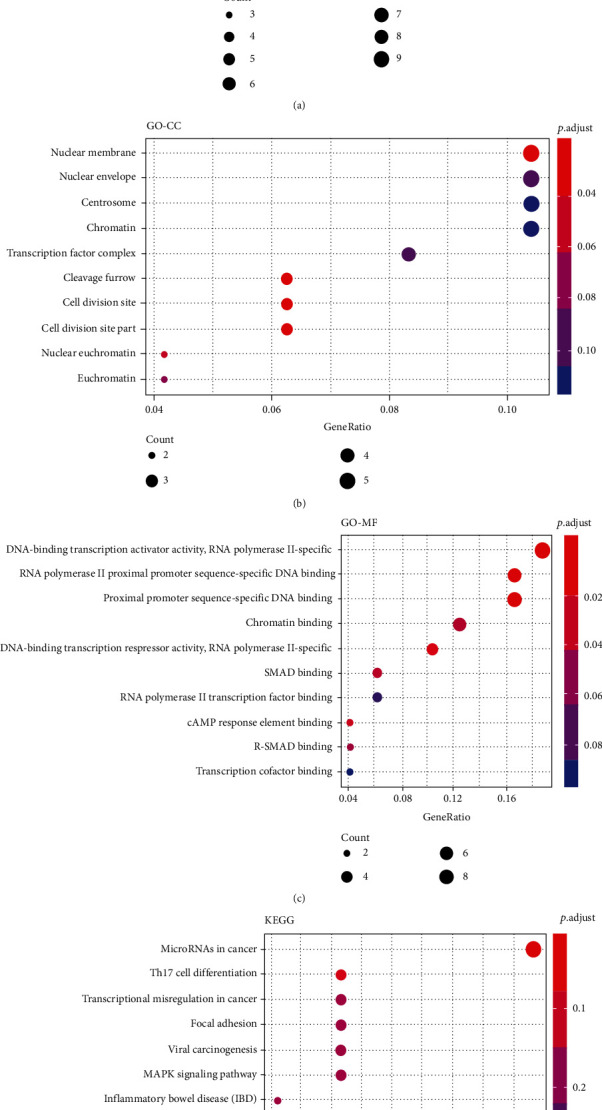
Functional enrichment analysis of the targeted mRNAs by GO and KEGG pathway analysis. (a) Results of the targeted mRNAs that were enriched in the biological process by GO analysis. (b) Results of the targeted mRNAs that were enriched in the cellular component by GO analysis. (c) Results of the targeted mRNAs that were enriched in the molecular function by GO analysis. (d) Results of the targeted mRNAs that were enriched in the metabolic pathway by KEGG pathway analysis.

We merged the differentially expressed circRNAs and miRNAs and then constructed the targeted interaction network with the overlapped mRNAs from TCGA and GEO databases to screen the hub genes with highly connectivity to each other. The differentially expressed circRNAs were uploaded into the search tool (http://bioinformatics.zju.edu.cn/Circ2Disease/search.html) for retrieving the targeted miRNAs. Then, two R packages (multiMiR and miRNAtap) were used to predict the targeted mRNAs of all differentially expressed miRNAs. We screened out the top 50 differentially expressed circRNAs, miRNAs, and mRNAs, respectively. We merged them to construct the targeted interaction network. Genes with highly interconnected nodes in the network were considered hub genes. In Figure 3, the green squares, red rhombuses, and orange dots represent circRNAs, miRNAs, and mRNAs, respectively. The size of them revealed the connection strength, showing that the bigger the size, the higher the connectivity they had. In the circRNA group, 18 hub circRNAs were identified, and the most remarkable genes were hsa_circRNA_104503/102682/105055/101525/100722/102002. For the miRNA group, about 24 hub miRNAs with high connectivity to each other were identified, such as hsa_miR_520e/429/3154/520b/137/5698. In the mRNA group, 51 hub mRNAs were identified with high connectivity. The top 10 remarkable genes with high connectivity were CDCA4, GATA6, LATS2, NR3C2, PDE5A, RAB23, RHOB, TMEM100, ZBTB4, and ZFPM2, which may be classified as candidate hub genes for further analysis. The biological functions of the differential targeted mRNAs (51 targeted genes) were explored, and functional analysis indicated that the most enriched GO-BP terms were the development of the eye, visual system, and sensory system (Figure 4(a)). The mainly enriched GO-CC terms were the nuclear membrane, nuclear envelope, centrosome, and chromatin (Figure 4(b)). For the GO-CC terms, the main enrichments were DNA-binding transcription activator activity and RNA polymerase II-specific and RNA polymerase II proximal promoter sequence-specific DNA binding (Figure 4(c)). Meanwhile, KEGG pathway analysis showed that the significantly enriched terms were related to the miRNAs in cancer, Th17 cell differentiation, and transcriptional misregulation in cancer (Figure 4(d)). The enrichment terms of these differentially expressed RNAs can be used to understand further the physiological functions of these hub genes.

### 3.4. Correlation Validation of the Central RNAs between the BCa and Normal Groups

**Figure 5:**
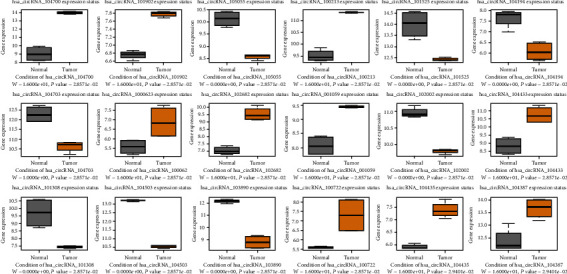
Expression profiles of the 18 hub circRNAs between bladder cancer and normal groups. The significant difference was under the condition of *P* value < 0.05.

**Figure 6:**
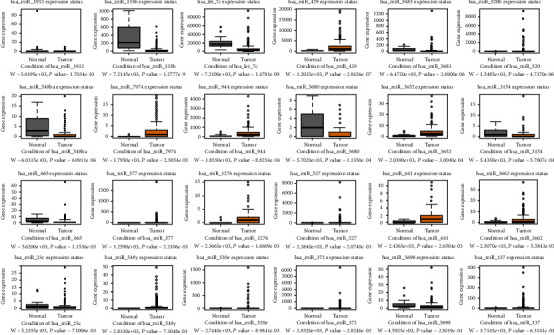
Expression profiles of the 24 hub miRNAs between bladder cancer and normal groups. The significant difference was under the condition of *P* value < 0.05.

**Figure 7:**
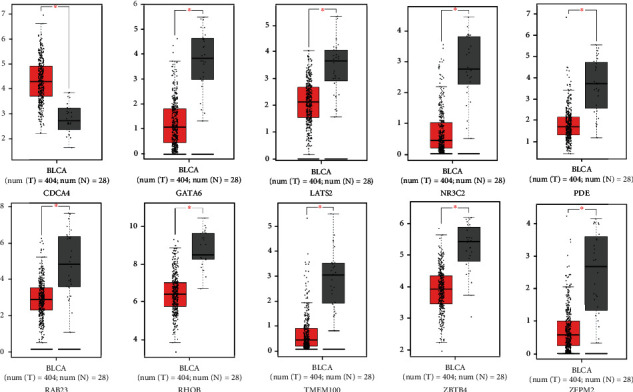
Expression profiles of the top 10 hub mRNAs between bladder cancer and normal groups. The significant difference was under the condition of *P* value < 0.05.

**Figure 8:**
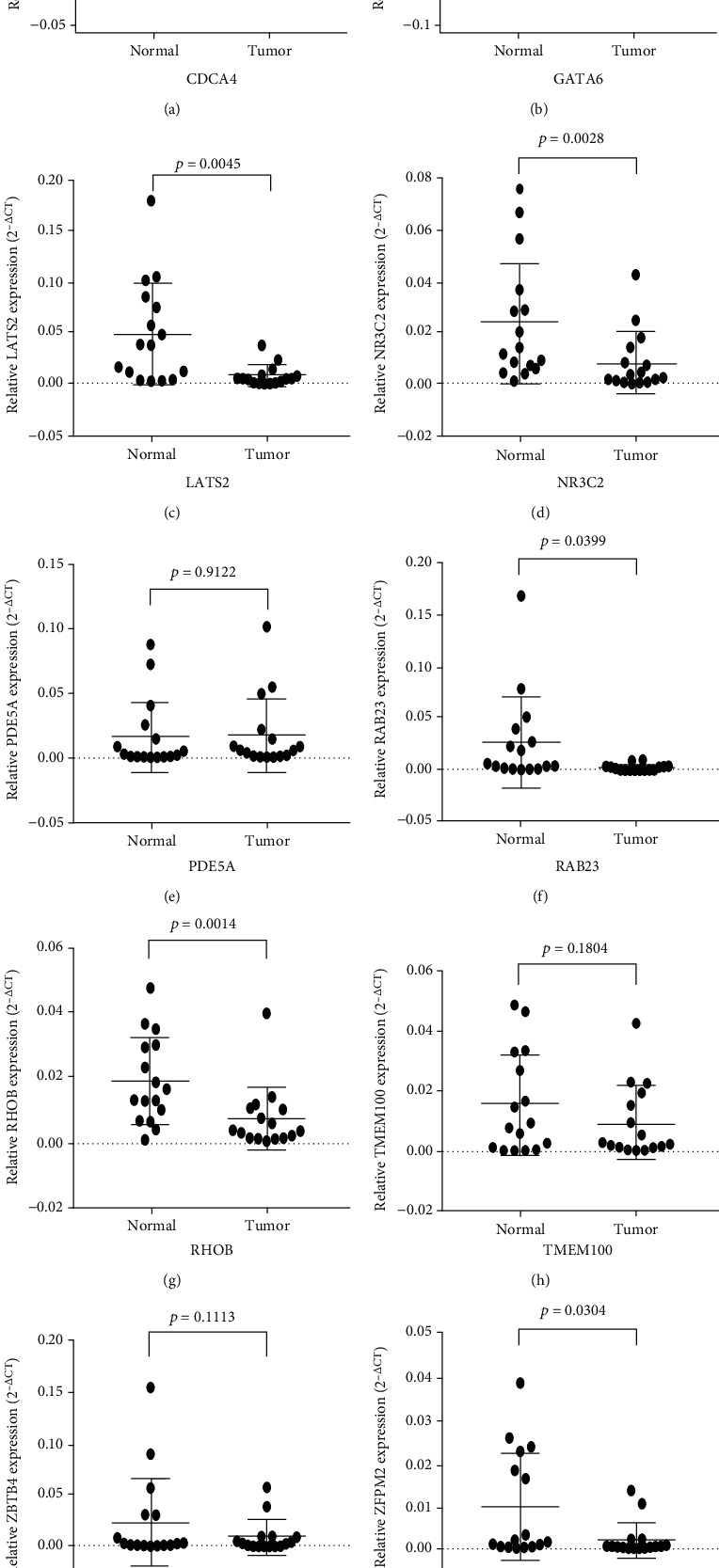
Expression validation of the top 10 hub mRNAs in 16 patients by qRT-PCR. The significant difference was under the condition of *P* value < 0.05.

We performed a differential expression analysis across the screening hub RNAs to provide an intuitive depiction of the central RNA expression profiles between the BCa and normal groups. For the central circRNAs, the expression levels of hsa_circRNA_104700/101902/100213/100062/102682/001059/104433/100722/104435/104387 significantly increased in the tumor samples. Meanwhile, the expression levels of hsa_circRNA_105055/101525/104194/104703/102002/101308/104503/103890 remarkably decreased in the tumor samples (Figure 5). In the miRNA groups, the expression levels of all central miRNAs, except hsa_miR_113a-2/133b/195/145/28/23b (downregulated), significantly increased in the tumor samples (Figure 6). For the top 10 targeted mRNAs, all of the hub genes, except CDCA4 (upregulated), were validated to be significantly downregulated in the BCa samples (Figure 7). We also validated the expression levels of these 10 mRNAs in 16 pairs of BCa tissues by using the qRT-PCR method. Our results are consistent with the previous analysis, except PDE5A (Figure 8). The protein levels of 10 mRNAs were verified by HPA analysis. The results showed a low expression level in BCa tissues compared with normal urinary bladder tissues except TMEM100 (Supplemental Figure 1).

### 3.5. Evaluation of the Prognostic Value of the Top 10 Hub mRNAs

**Figure 9:**
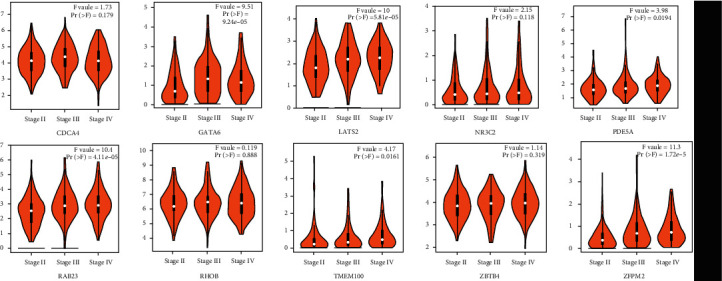
Correlations of top 10 hub genes between expressions and tumor stages. The significant difference was under the condition of *P* value < 0.05.

The statistical significance of the expression distribution in diverse tumor stages was calculated using one-way ANOVA to explore the prognostic value of the 10 hub genes. In this study, a low stage (tumor stages I and II) and high stage (tumor stages III and IV) were selected for the expression analysis. The GATA6, LATS2, PDE5A, RAB23, TMEM100, and ZFPM2 genes showed significantly positive correlations between expression and stages (*P* < 0.05) (Figure 9). Thus, the regulatory roles of these genes in tumor progression are worthy of further exploration.

**Figure 10:**
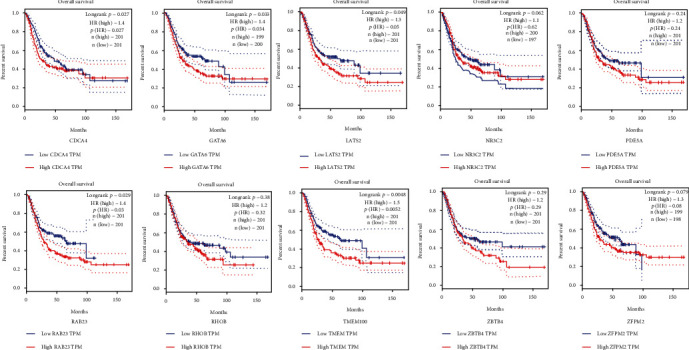
Results of the overall survival analysis of the top 10 hub genes in bladder cancer. The significant difference was under the condition of *P* value < 0.05.

Furthermore, the overall survival analysis was performed via the Kaplan–Meier curve test to verify the regulatory roles of these hub genes. Results showed that the hub genes outstanding in the current survival analysis were CDCA4, GATA6, LATS2, RAB23, and TMEM100 (*P* < 0.05). The other genes had no significant correlation in overall survival (Figure 10).

### 3.6. Validation of Hub Gene Expression across Cancers

**Figure 11:**
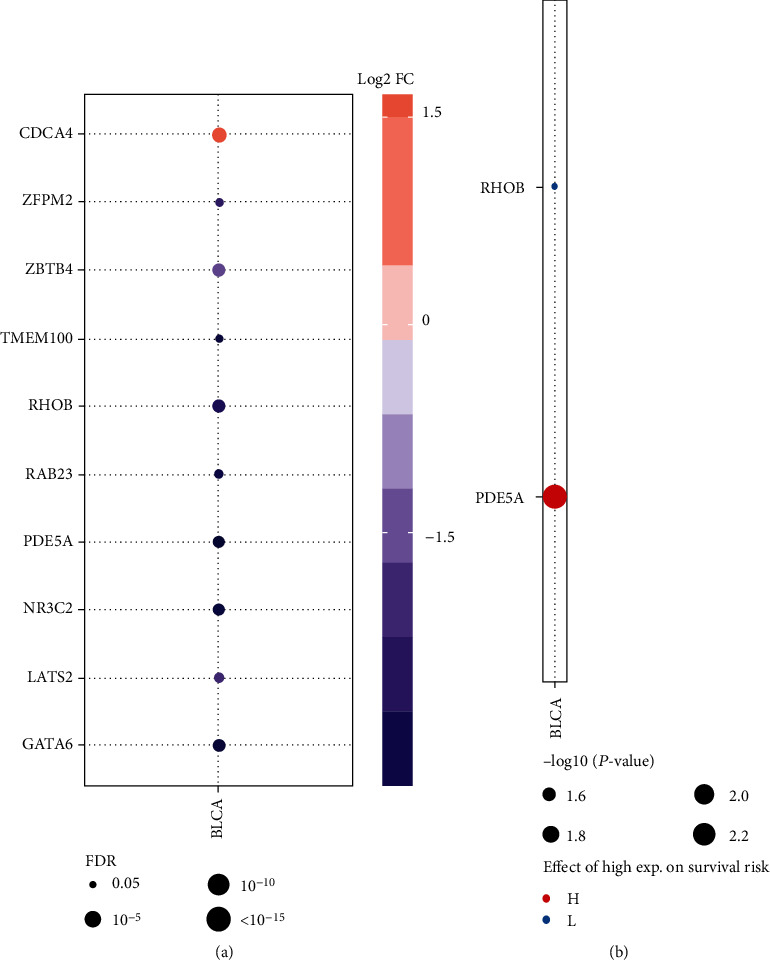
Expressions of the hub genes across cancers. (a) Results of the hub gene expression between tumor and normal groups. (b) Results of the hub gene expression in the survival with diverse cancer types. The significant difference was under the condition of *P* value < 0.05.

TCGA database provides rich clinical follow-up information, including the use of drugs, relapse, and survival in diverse cancers. Exploring the expression profiles across cancers helped in elucidating the regulatory roles of the hub genes. Results showed that all of the genes, except CDCA4 (positive), played a negative role across cancers (Figure 11(a)). Moreover, the PDE5A gene showed the highest correlation with survival risk, whereas the RHOB gene had the lowest correlation in diverse cancer types (Figure 11(b)).

### 3.7. Pathway Activity of the Hub Genes Associated with BCa

**Figure 12:**
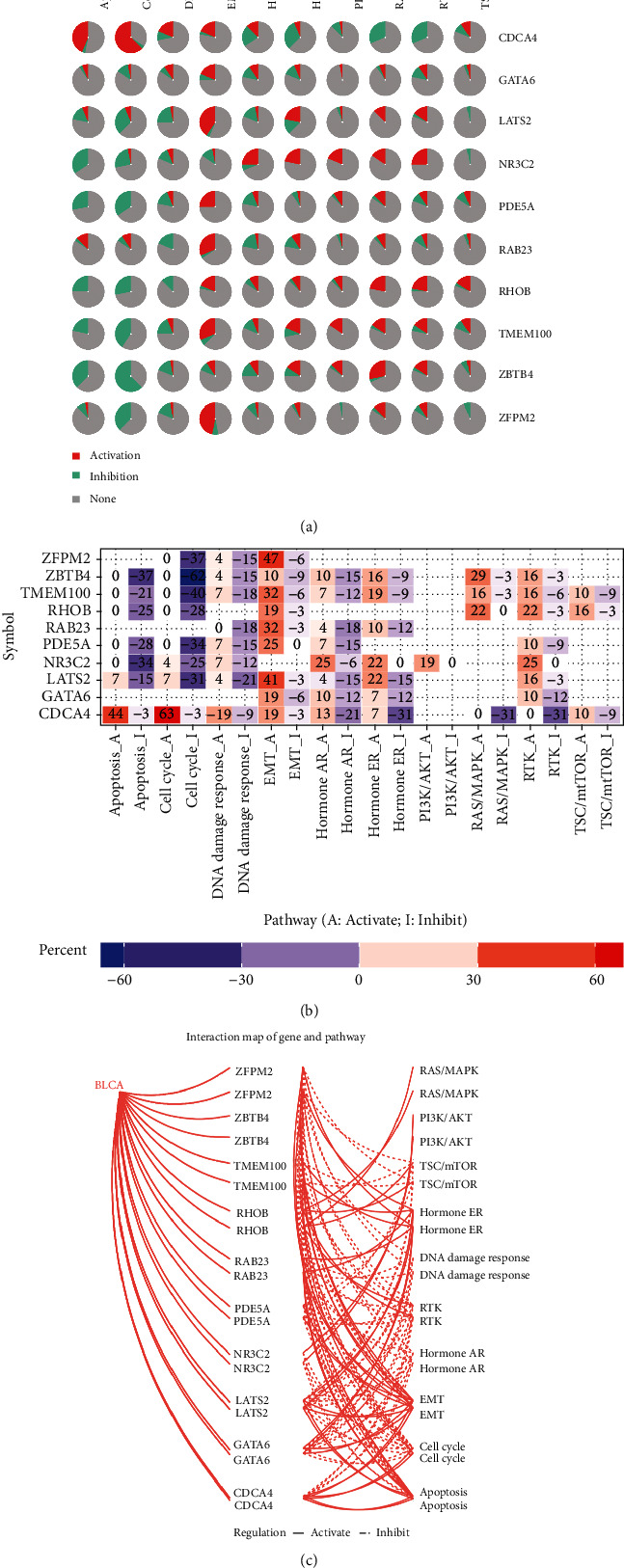
Pathway activity analysis of the hub genes. (a) Global percentage of the hub genes in activating or inhibiting pathways. (b) Heat map of the hub genes with percentage scores. (c) Relation network of the hub genes and pathways.

The regulatory roles of the hub genes in pathological mechanisms were explored. Ten classical pathways associated with cancers were selected to evaluate the pathway activity regulated by each hub gene. Results indicated that all of the pathways may be activated by these genes in varying degrees. The pathways of apoptosis, cell cycle, and DNA damage response were mainly inhibited by most hub genes, except CDCA4, which showed an activation role. Meanwhile, the EMT pathway was outstanding for its activation across the hub genes. Other pathways, such as hormone ER, PI3K/AKT, RAS/MAPK, and RTK, were activated by most genes (Figures 12(a) and 12(c)). In addition, heat map percentage analysis indicated that the top three outstanding pathways were EMT (activation), cell cycle, and apoptosis (inhibition) with high scores across these hub genes (Figure 12(b)).

### 3.8. Correlations between Hub Genes and Clinical Outcomes

**Figure 13:**
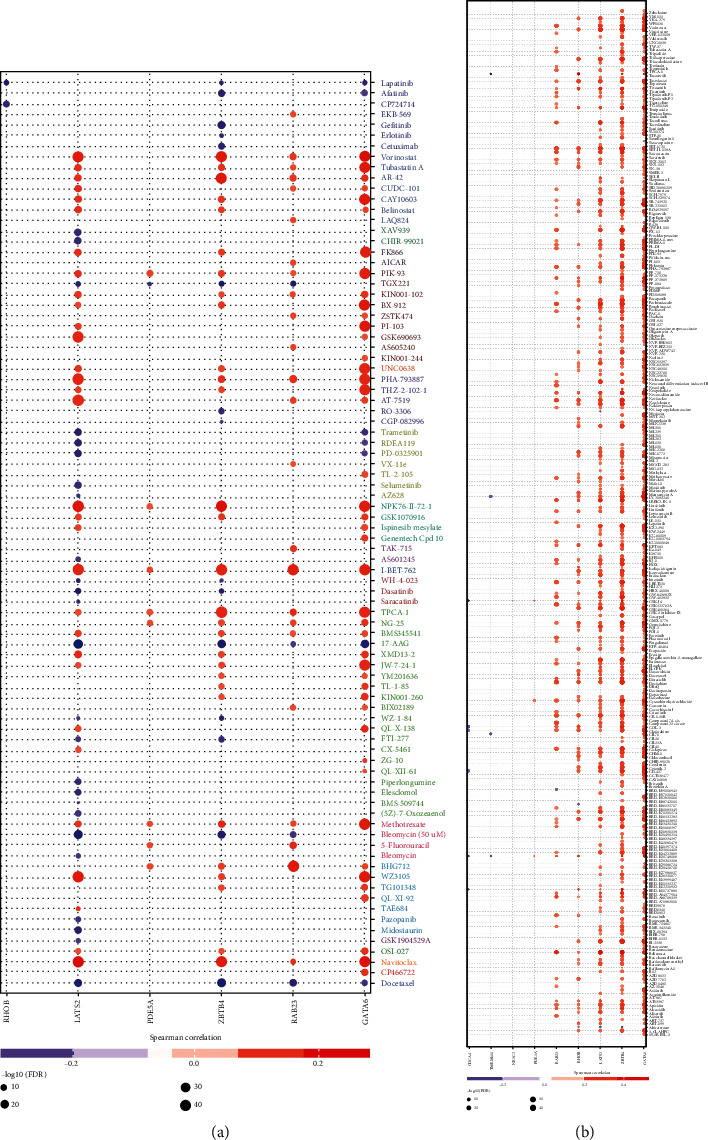
Results of drug sensitivity analysis of the outstanding hub genes.

We performed a drug sensitivity analysis to evaluate the correlation of hub genes and clinical outcomes. Results indicated that the most outstanding genes were GATA6, ZBTB4, and LATS2, which were connected with drugs and small molecules in the GDSC and CTRP groups; these genes were then followed by RAB23 and RHOB (Figure 13). Moreover, the most efficient drugs/small molecules that were connected with hub genes were vorinostat, tubastatin A, NPK76-II-72-1, I-BET-762, navitoclax, WZ3105, PHA-793887, TPCA-1, and so on in the GDSC groups (Figure 13(a)). In the CTRP groups, the most efficient drugs/small molecules were vorinostat, SR-II-138A, panobinostat, PHA-793887, belinostat, SR-II-138A, CR-1-31B, and so on (Figure 13(b)). These results revealed that these hub genes were significantly correlated with clinical outcomes, and they may have potencies as drug targets and biomarkers of BCa.

## 4. Discussion

BCa is the most common malignant tumor of the urinary system [1]. Given its high incidence and complex etiology, BCa became one of the top 10 malignant tumors with high mortality in the world [1, 2]. Targeted therapy is a new type of strategic therapy for BCa [6]. Compared with immunotherapy, it is characterized by its capacity to specifically target cancer cell growth-related mutant genes, message transmission pathways, or growth factor receptors to suppress or destroy tumor cells, thereby suppressing or eliminating tumors [6, 16]. Biomarkers that can be used as effective candidate genes in BCa therapy remain unclear because of the extensive and complex mutual assistance networks of targeted genes. RNA is an important regulatory factor for gene expression; it plays a crucial role in tumor genesis, progression, and prognosis [9, 17]. Although RNA has been extensively studied in recent years, its specific mechanism of action in BCa has not yet been clarified, and further research is needed.

In the present study, we first systematically analyzed the differentially expressed RNAs of circRNAs, miRNAs, and mRNAs in BCa. GO and KEGG enrichment analyses were performed to reveal the potential regulatory roles of these differentially expressed RNAs in BCa. Results showed that they were mainly enriched in the GO terms of muscle and skeletal system development, DNA transcription translation, and channel activity. Skeletal muscle not only is the driving force of exercise but also is the key regulator of the entire body's metabolism, which might be driven by cancer-related factors [18, 19]. During transcription, the abnormal transcription factors or gene mutations could affect gene expression, including blocking cell differentiation and death programs, which are a hallmark for cancer [20]. In addition, the enriched signaling pathways of calcium, cAMP, cGMP-PKG, and even the ligand-receptor interaction are all involved in tumor procession and prognosis [21–23]. These results suggest that differential RNAs play important roles in BCa.

Many studies have shown that dysregulation of circRNA expression is related to the pathogenesis of BCa, tumor progression, and prognosis and can be used as biomarkers for BCa [24, 25]. Li et al. revealed that circCdr1as is significantly downregulated in BCa tissues and cell lines. Overexpression of circCdr1as could inhibit the proliferation, invasion, and migration of BCa cells in vitro and slow down the growth of tumors in vivo. The study also found that circCdr1as could directly target miR-135a and inhibit its activity to exert anticancer effects [24]. Similarly, circHIPK3 could target miR-558 for sponging to inhibit the expression of heparanase (HPSE) and suppress the migration, invasion, and angiogenesis of tumor cells [25]. Many studies have found that miRNA also plays an important role in the development of tumors, and miRNA may become an important target for the early diagnosis and treatment of bladder cancer [26, 27]. Taheri et al. revealed that aberrant expression of miRNAs may also provide new diagnosis biomarkers in bladder cancer [26]. Ding et al. screened out a group of miRNAs related to the progression of bladder cancer by bioinformatics methods and also provided a new direction for the diagnosis and treatment of bladder cancer [27]. In the present study, we generated a global triple interaction network based on circRNA-miRNA and miRNA-mRNA targeting prediction. The network (circRNA-miRNA-mRNA) was composed of 18 circRNAs, 24 miRNAs, and 51 mRNAs. In this network, hsa_circ_104503 and 102682 were the most outstanding circRNAs that target with the maximum number of miRNAs, which were rarely identified in BCa and other diseases. However, the most remarkable miRNAs were has_miR_520e/429/3154/520b/137 with high connectivity with diverse mRNAs in this network. These central miRNAs were also rarely identified in BCa but associated with other cancers. For example, hsa_miR_520e expression is downregulated in breast cancer tissues and might promote cell migration and apoptosis in vitro [28]. The hsa_miR_429 is downregulated and could reduce the migration ability of HCC cells by targeting the RAB23 gene [29]. Meanwhile, hsa_miR_137 could directly target with the PIK3R3 gene and inhibit its function, thereby suppressing the migration and invasion of tumor cells [30]. Given these relevant evidence, the central circRNAs and miRNAs were speculated to be potential biomarkers for BCa treatment.

We identified 10 hub genes that were the top 10 outstanding mRNAs (CDCA4, GATA6, LATS2, NR3C2, PDE5A, RAB23, RHOB, TMEM100, ZBTB4, and ZFPM2) in the targeted interaction network. The expression levels of these hub genes, except for CDCA4, significantly decreased in BCa tissues; CDCA4 showed a remarkably upregulated role in the tumor. In our own patients, the results are consistent with the previous analysis except PDE5A, TMEM100, and ZBTB4. Moreover, among them, six hub genes (GATA6, LATS2, PDE5A, RAB23, TMEM100, and ZFPM2) were significantly correlated with the pathological stage, whereas five of them (CDCA4, GATA6, LATS2, RAB23, and TMEM100) had significantly poor prognosis in BCa. GATA6 belongs to the transcription factor family that associated with many diseases [31]. In BCa, low expression of GATA6 could promote lymph node metastasis, which might serve as a predictor of early recurrence and short survival [32]. It is directly targeted by has-miR-944 that binds with has_circRNA_105055 and might serve as a suppressor in BCa. LATS2 is a Dbf2-related kinase that acts as a central regulator of cell fate by regulating the function of numerous carcinogenic factors or tumor suppressor effectors [33]. In non-small-cell lung cancer, low expression of LATS2 may result in poor prognosis [34]. Our finding of LATS2 was consistent with its correlation with tumor stage and poor prognosis. LATS2 was targeted by has-miR-372 that binds with has_circRNA_104503 and 102002. It also has high scores in the activation of the ENT pathway and inhibition of the cell cycle, which are closely related to tumorigenesis and progression [35, 36]. Thus, the LATS2 mRNAs may serve as suppressors of anti-BCa. PDE5A acted as an oncogenic factor in melanoma cells. Its downregulation would result in increased cGMP and cytosolic Ca^2+^, thereby increasing contractility and inducing tumor cell invasion [37]. In the present study, PDE5A was downregulated in the BLCA group. It showed a high score in inhibiting cell cycle transition, which indicated that PDE5A may play a negative role in BCa. But the survival risk analysis of PDE5A was not significant. RAB23 belongs to the RAB subfamily and is a key regulator of cell membrane trafficking. It is a tumorigenic or metastatic biomarker for many cancers [38]. We found that the RAB23 played a negative role across cancers and had a high score in activating the EMT pathway, which might enhance the invasiveness of tumor cells and generate circulating tumor cells [36]. TMEM100 or transmembrane protein 100 contains two hypothetical transmembrane domains and is associated with tumor progression [39, 40]. Its low expression could promote tumor cell proliferation, invasion, and migration, leading to a poor prognosis in non-small-cell lung carcinoma [40]. Although in the BLCA group, TMEM100 has a high expression level in the normal group, the verification of it in our own patients was insignificant. And the survival analysis showed that TMEM100 was negatively associated with survival time of patients in BLCA. Therefore, the role of TMEM100 in BCa needs to be further explored. CDCA4 or cell division cycle-associated protein 4 is related to tumor cell proliferation and apoptosis. In breast cancer, transfecting with CDCA4 in vitro could enhance the proliferation and reduce the apoptosis of MCF-7/ADM cells [41]. In the present study, CDCA4 was targeted by has-miR-548ba that binds with has_circRNA_100722. Its remarkable activations on cell cycle and apoptosis suggest that it can serve as a promotor of BCa. These results revealed that the outstanding hub genes may play important roles in regulating tumor cell proliferation, metastasis, and apoptosis, which make them potential biomarkers for cancer diagnosis and prognosis.

Drug sensitivity analysis showed that the outstanding genes of GATA6, ZBTB4, and LATS2 were sensitively connected with a large number of drugs and small molecules, such as vorinostat, PHA-793887, belinostat, SR-II-138A, and panobinostat. Vorinostat is a histone deacetylase inhibitor widely used in cancer treatment. It could upregulate the MICA via the PI3K/Akt pathway to enhance the lethality of immune cells to tumors, thereby achieving anticancer effects [42]. PHA-793887 is an inhibitor of multiple cyclin-dependent kinases (CDK) with activity against CDK2, CDK1, and CDK4 [43]. Belinostat is a histone deacetylase inhibitor that may inhibit cell proliferation via the Wnt/*β*-catenin pathway and induce cell apoptosis in breast cancer [44]. Panobinostat is a pan-deacetylase inhibitor that could disrupt the protein biological function by interfering with the enzymatic activity of deacetylase [45]. In conclusion, these hub genes that are highly connected with drugs and small molecules may provide a basis for the clinical treatment and guide the clinical medication of BCa.

Studies have reported that many environmental factors are important causes of BCa, and exposure to these environmental pollution factors causes influences in many important pathways [46]. Not only mRNAs but also many circRNAs, lncRNAs, and miRNAs will be affected by environmental factors, and the pathways they lead or participate in will also be inhibited or promoted, thus causing the occurrence and development of bladder cancer [47–49]. In this study, we identified some potential circRNA-miRNA-mRNA pathways which may inhibit the tumorgenesis and progression of BCa, while the environmental carcinogens and other harmful stimuli may destroy the stability of these tumor suppressor pathways and then induce the occurrence or progression of BCa.

## 5. Conclusion

We conducted a comprehensive analysis of circRNAs, miRNAs, and mRNAs differentially expressed in BCa and constructed a circRNA-miRNA-mRNA targeting interaction network. The central RNAs with highly connected nodes were identified in this network. Then, functional enrichment, tumor progression, and correlation analyses of prognosis were performed to explore the regulatory roles of related hub genes in BCa. Drug sensitivity analyses were conducted to verify the biological functions of these hub genes. This study reveals the regulatory function of the circRNA-miRNA-mRNA chain in BCa and provides potential therapeutic targets for BCa.

## Figures and Tables

**Table 1:** Results of the differential expression analysis among the GSE92675, GSE133624, and TCGA BLCA datasets.

Type	Comparison	Log_2_FC_cutoff	FDR_cutoff	All gene_num	Up gene_num	Down gene_num
circRNA	Tumor vs. normal	1	0.05	434	267	167
miRNA	Tumor vs. normal	1	0.05	367	269	98
mRNA (GEO)	Tumor vs. normal	1	0.05	4799	1903	2896
mRNA (TCGA)	Tumor vs. normal	1	0.05	4841	2717	2124

**Table 2:** Results of the differential overlapped mRNAs from the merging datasets of GSE133624 and TCGA BLCA.

Type	Comparison	Log_2_FC_cutoff	FDR_cutoff	All gene_num	Up gene_num	Down gene_num
mRNA	Tumor vs. normal	1	0.05	2290	971	1319

## Data Availability

All data generated or analyzed during this study are included in this published article.

## Supplementary Materials

Supplementary Materials Supplemental Table 1: all PCR primers used in this research. Supplemental Figure 1: protein level validation of the top 10 hub genes by HPA analysis.
