# Supplementary material for: Identification of the circRNA-miRNA-mRNA Regulatory Network in Bladder Cancer by Bioinformatics Analysis
Source: Int J Genomics. 2021 Nov 16;2021:9935986. doi: 10.1155/2021/9935986 (PMC8610721; doi:10.1155/2021/9935986)
Supplement: Supplementary Materials — Supplemental Table 1: all PCR primers used in this research. Supplemental Figure 1: protein level validation of the top 10 hub genes by HPA analysis. [file 9935986.f1.docx]

**Table 1:** All PCR primer used in this research

| Primers and probes |  | sequence |
| --- | --- | --- |
| CDC4 | Forward  Reverse | 5’-CCCTGAAACAGCGTTGGGAA-3’  5’-CGGATGAACGATCCCTTTAGC-3’ |
| GATA6 | Forward  Reverse | 5’-CTCAGTTCCTACGCTTCGCAT-3’  5’-GTCGAGGTCAGTGAACAGCA-3’ |
| LATS2  NR3C2  PDE5A  RAB23  RHOB  TMEM100  ZBTB4  ZFPM2  β-actin | Forward  Reverse  Forward  Reverse  Forward  Reverse  Forward  Reverse  Forward  Reverse  Forward  Reverse  Forward  Reverse  Forward  Reverse  Forward  Reverse | 5’-ACTTTTCCTGCCACGACTTATTC-3’  5’-GATGGCTGTTTTAACCCCTCA-3’  5’-GAAAGACGGTGGGGTCAAGTT-3’  5’-ACCGGAAACACAGCTTACGTT-3’  5’-GCAGAGTCCTCGTGCAGATAA-3’  5’-GTCTAAGAGGCCGGTCAAATTC-3’  5’-TGGTGGTTGTAGGGAATGGAG-3’  5’-CTGTGGTAGAGAACACGAGCA-3’  5’-CTGCTGATCGTGTTCAGTAAGG-3’  5’-TCAATGTCGGCCACATAGTTC-3’  5’-TGCTGTGGTTGTCTTCATCG-3’  5’-CTCTCCCGTCTCTTGGCTTTC-3’  5’-TTGCTGTCGGCAGTAGTTATTT-3’  5’-GTGTTACGTGATCCAGGGTGA-3’  5’-ATGTCCCGGCGAAAGCAAA-3’  5’-AGCTCAGATTTTCAGGCCCAA-3  5’-CTCCATCCTGGCCTCGCTGT-3’  5’-GCTGTCACCTTCACCGTTCC-3’ |
